# Boerhaave's syndrome with late presentation. Experience in an Argentine single center: Case series

**DOI:** 10.1016/j.amsu.2019.07.023

**Published:** 2019-07-13

**Authors:** Daniel N. Velasco Hernández, Héctor R. Horiuchi, Lucas A. Rivaletto, Fidelio Farina, Matías Viscuso

**Affiliations:** University Hospital General San Martín, La Plata, Buenos Aires, Argentina

**Keywords:** Boerhaave's syndrome, spontaneous esophageal rupture, esophageal perforation

## Abstract

**Background:**

In the year 1724, Hermann Boerhaave reported a case of a Dutch admiral who died due to spontaneous rupture of the esophagus following vomiting. The aim of this retrospective study is to analyze the therapeutic modality, morbidity and mortaliy of a group of patients with spontaneous esophageal rupture treated in our hospital.

**Methods:**

Ten patients were treated from March 1990 to August 2016. Seven patients were males and three were females. The age range was, 52–72 years, with an average of 66.2 years. In four patients, the diagnosis and posterior treatment were performed within 24 h (Group I) and the remaining six patients after 24 h (Group II).

**Results:**

The mean hospital stay was 36.6 days (range 17–62 days). The mortality rate was 50%, which was due to septic shock and the morbidity of patients who survived was 40% due to pneumonia in one case and fistula in another.

**Discussions:**

This condition has a high mortality rate with a lethality that depends on the time between recognition of symptoms and proper surgical treatment.

## Highlights

• Boerhaave's syndrome consists of a transmural laceration occurring most frequently at the left posterolateral aspect of the distal esophagus due to a sudden increase intraluminal pressure.
• The typical signs of this condition are: pain, vomiting, dyspnea and shock. Esophagogram an CT scan are the most frequent tools used for diagnosis due to its 75% sensitivity.
• Most of the reports in the literature agree with performing primary closure within the first 24 h. Due to its high failure rate, primary closure is not advised if performed after 24 h.

## 1 Introduction

In the year 1724, Hermann Boerhaave reported a case of a Dutch admiral who died due to spontaneous rupture of the esophagus following vomiting. He also gave a detailed description of the symptoms and the pathophysiological findings defining the condition known as Boerhaave's syndrome [[1], [2], [3]].

This condition consists of a transmural laceration occurring most frequently at the left posterolateral aspect of the distal esophagus due to a sudden increase intraluminal pressure. Given that this condition is difficult to diagnose and that it is considered one of the most lethal gastrointestinal perforations, it is a topic under continuous revision in the literature [4,5].

The purpose of this retrospective study is to analyze a group of patients with spontaneous esophageal rupture by considering the time elapsed until reaching proper diagnosis for the choice of surgical treatment, and the morbidity and mortality rate.

## 2 Patients and methods

From March 1990 through March 2016, 10 patients were treated for spontaneous rupture or Boerhaave's syndrome. Of these, seven were males and three were females. The age range was, 52–72 years, with an average of 66.2 years.

The frequent clinical symptoms included: vomiting, thoracic pain, epigastric pain, emphysema and hematemesis. Mackler triad (vomiting, thoracic pain and subcutaneous emphysema) was observed in only three cases (30%) [6,7]. The characteristics of the patients and the symptoms are presented in Table 1.

**Table 1:** Patients characteristics and presenting symptoms.

Characteristic	Data^a^
Age	66.2 (52–72)
Sex (M: F)	7:3
Background	
Tobacco	10
Alcoholic	8 (7 males and 1 female)
Obesity	4 (3 males and 1 female)
Symptoms	
Vomiting	90% (9)
Thoracic pain	70% (7)
Epigastric pain	30% (3)
Emphysema	20% (2)
Hematemesis	10% (1)
Time to diagnosis	
<24 h (Group I)	4
>24 h (Group III)	6

a Data are shown as number, % or median (range).

In all cases, laboratory findings evidenced leukocytosis, and in 80% of the cases, thorax Rx evidenced some alteration (left pleural effusion in six cases, pneumomediastinum in two and mediastinal thickening in one). Esophagogram and thoracoabdominal CT scan confirmed the diagnosis, although one case required video esophageal endoscopy.

The rupture was always evident at the left side of the distal esophagus.

Data were collected from the clinical and surgical records. That the work has been reported in line with the process criteria [8].

## 3 Results

All the patients were submitted to the Intensive Care Unit. Three Group I patients underwent suture and mediastinal drainage, and only one required suturing plus cardioligature with cervical esophagostomy known as esophageal exclusion. In Group II, three patients underwent pleural lavage and drainage plus esophageal exclusion, one patient required drainage of the perforation with a T-tube due to closure difficulty caused by necrosis, one underwent transhiatal esophagectomy, and one only underwent pleural drainage due to critical condition. Except for this latter patient, all the other patients underwent feeding jejunostomy for nutritional support.

The surgical procedures and mortality are summarized in Table 2.

**Table 2:** Surgical procedures and mortality.

Group	N	Surgical Procedures (Number)	Mortality
I (<24 h)	4	Suture and drainage (3)	0%
		Esophageal exclusion (1)	
II (>24 h)	6	Esophageal exclusion (3)	83.3% (5)
		Esophagectomy (1)	
		Perforation drainage (1)	
		Pleural drainage (1)	
			Total 50% (5)

The mean hospital stay was 36.6 days (range 17–62 days), and the morbidity of patients who survived was 40% due to pneumonia in one case and fistula in another, with consequent need for surgical intervention for washing and drainage. Both patients were in Group I. The overall mortality was 50%, and the difference between Group I and Group II was important.

## 4 Discussion

According to different literature reports, spontaneous esophageal rupture or Boerhaave's syndrome represents 10–40% of esophageal perforations. It usually manifests with greater frequency in middle aged male patients, with a history of alcohol use. The condition is considered of high mortality risk, which depends of the time elapsed between symptom manifestation and proper surgical treatment [9,10].

The physiopathology of this condition is characterized by a sudden increase in intraesophageal pressure, which is frequent during vomiting, causing neuromuscular incoordination due to failure of cricopharyngeal relaxation. This situation along with the absence of mucous muscularity in the left distal esophagus causes parietal disruption, allowing saliva, food remains and gastric juices to enter into the mediastinum and/or pleura [10,11].

According to Brauer et al., the typical signs of this condition are: pain (83%), vomiting (79%), dyspnea (39%) and shock (32%) [12].

Usually, cases that are reported include middle aged male patients with previous alcohol consumption. These cases, after gluttonous intake, present with vomiting, thoracic or epigastric pain, dyspnea, pneumomediastinum, subcutaneous emphysema and, finally, sepsis and shock. The typical Mackler triad (vomiting, thoracic pain and subcutaneous emphysema) is evident only in half of the cases [6].

The differential diagnosis of esophageal perforation is quite broad. The first step is to consider the possibility of its existence due to potentially lethal thoracic pain. Among other entities perforating the ulcer, myocardial acute infarct, aortic dissection, acute pancreatitis and pulmonary embolism should be disregarded. Thoracic X-ray shows distortions in 90% of the cases, thus demonstrating pneumomediastinum, hydropneumothorax, subcutaneous emphysema and mediastinal thickening [7].

Esophagogram is the most frequent tool used for diagnosis due to its 75% sensitivity. Consequently, a normal study does not disregard the diagnosis. CT scan is helpful in severe cases where patients are incapable of cooperating during the contrastive study and in cases without evidence of perforation. Moreover, it allows the evaluation of structures like the pleura, mediastinum and aorta, although it does not allow for the determination of the exact level of the lesion's location [[11], [12], [13]].

For this condition, surgery is the treatment of choice on addition to principally bearing in mind the time elapsed from the detection of the condition and the selection of an appropriate technique [14,15].

Most of the reports in the literature agree with performing primary closure within the first 24 h. It consists of a simple closure or using a pleural or diaphragmatic patch for suture closure reinforcement. Due to its high failure rate, primary closure is not advised if performed after 24 h [16]. Lawrence published nine cases, of which only one died after primary closure was performed within 24 h after the rupture [17]. For late diagnosis, septic focus drainage, cervical esophagostomy and jejunostomy for nutritional support are the standard treatments of choice. There are reports that confirm some good results obtained with endoscopic treatment and the placement of expandable prosthesis or clips also using minimally invasive surgery for repair or only drainage [[18], [19], [20]]. Endoluminal Vacuum therapy is another option that be utilized in critically ill individuals, early and late perforations. Although disadvantages of this method of treatment are prolonged hospital stay and the need for multiple endoscopic procedures [21]. Considering the surgical team experience, the initial esophagectomy may be considered a valid procedure. The prevalence of high mortality in our series was mainly due to delayed presentation of symptoms and, subsequently, delayed diagnosis [22,23].

We conclude that spontaneous esophageal rupture should be included within the differential diagnosis of every patient with acute thoracic pain and vomiting, since the non-specificity of its symptoms often leads to an erroneous presumptive diagnosis and late confirmation.

## Ethical approval

The ethical approval has been exempted by the institution.

## Sources of funding

The sources of financing were their own and without sponsors.

## Author contribution

Specific contributions of authors.

Dr. Horiuchi: Acquisition of data and critical revision.

Dr. Viscuso: Analysis and interpretation of data.

Dr. Farina: Study conception and design and bibliography search.

Dr. Rivaletto: Drafting of manuscript and bibliography search.

## Conflicts of interest

No conflict of interest.

## Trial registry number

researchregistry4384.

## Guarantor

Norberto Daniel Velasco hernández.

## Consent

Written informed consent was obtained from the patient for publication of this cases series. We do not send images or personal data of patients.

## Provenance and peer review

Not commissioned, externally reviewed.
